# Factors Contributing to the Intention to Leave Among Nursing Home Staff in Singapore: A Cross‐Sectional Study

**DOI:** 10.1155/jonm/3338222

**Published:** 2025-12-29

**Authors:** Hongli Sam Goh, Swee Jek Sim, Mun Leong Tang, M. Kamala Devi

**Affiliations:** ^1^ St. Andrew’s Mission Hospital, Singapore; ^2^ Graduate School, Charisma University, Grace Bay, Turks and Caicos Islands; ^3^ School of Medicine, Dentistry and Nursing, University of Glasgow, Glasgow, UK, gla.ac.uk

**Keywords:** healthcare, human resource management, nurses, nursing home, retention, staff attrition

## Abstract

**Introduction:**

Nursing home staff turnover remains a concern in long‐term care facilities, with implications for job performance, organisational morale and patient outcomes. This study explored the factors influencing intention to leave among nursing home care staff in Singapore and focused on the influence of demographic variables, work environment and job satisfaction.

**Methods:**

A cross‐sectional survey was employed to collect data from 167 care staff working at two nursing homes in Singapore. Instruments included the Practice Environment Scale of the Nursing Work Index (PES‐NWI), Job Satisfaction Survey (JSS) and Intention to Leave Scale (ILS). Data were analysed using descriptive and inferential statistics, including hierarchical multiple regression, to examine relationships between demographic characteristics, work environment, job satisfaction and intention to leave.

**Results:**

The study found a moderate overall intention to leave (mean ILS = 9.18, SD = 2.494). Female staff, mid‐career professionals (aged 30–39) and nursing aides exhibited the highest turnover intentions. Job satisfaction, particularly in terms of managerial support and staffing adequacy, emerged as a significant predictor of intention to leave. Work environment factors, especially staffing and resource adequacy, are crucial in turnover intentions.

**Discussion:**

This study highlights key factors driving turnover intentions among nursing home care staff in Singapore. Interventions to mitigate turnover and improve staff retention are recommended to improve staffing levels, managerial support and career development pathways.

## 1. Introduction

The turnover rate among nurses and unlicensed assistive personnel (e.g., nursing aides and healthcare assistants) remains a concerning global workforce issue. The high attrition rate among nurses and unlicensed assistive personnel continues to draw the attention of nurse researchers as it can severely impact healthcare organisations through reduced staff performance, lower morale, poorer patient outcomes and a domino effect on additional turnover [1, 2]. By understanding the factors that drive nursing turnover, nurse leaders would be able to improve their organisations’ retention rates [3, 4]. Although many studies have been conducted to examine turnover among nurses, revealing a variety of factors’ driving turnover rate [5, 6], few studies focused on unlicensed assistive personnel despite them serving as the majority of the aged care workforce within the long‐term care sector [7]. Thwaites et al. [7] stated that the annual median turnover rate for nursing assistants could be as high as 99% in the United States. In Singapore’s long‐term care sector, unlicensed assistive personnel form the backbone of the workforce. Nursing aides are typically foreign‐trained nurses whose qualifications are not recognised locally or who lack the recent clinical experience required for licensure, whereas healthcare assistants possess foundational care training and support residents with daily routines [8]. Despite their critical role, turnover rates among both nurses and unlicensed assistive personnel in Singapore remain poorly understood, highlighting the need for focused research.

As intention to leave is a strong predictor of actual turnover, understanding the various factors influencing the intention to leave among nurses and care staff is essential. Alenazy et al. [9] emphasised that gaining insights into these factors can enable management and supervisors to develop effective strategies to improve retention and mitigate actual turnover. These strategies can also enhance job satisfaction and foster a healthier work culture and environment. Factors influencing the intention to leave can be multifaceted, encompassing job satisfaction, organisational support, personal circumstances, work‐related stress and remuneration [5, 9, 10]. Among these factors, job satisfaction plays a key role in turnover intent and is influenced by several elements, including the work environment, workload and opportunities for career development. When staff members feel supported by their organisation and experience effective leadership, they are more likely to remain in their positions [11]. Conversely, high levels of work‐related stress, burnout and inadequate work‐life balance can significantly increase the intention to leave. Furthermore, competitive salaries and benefits are crucial in retaining both nurses and care staff [2].

**Figure 1:**
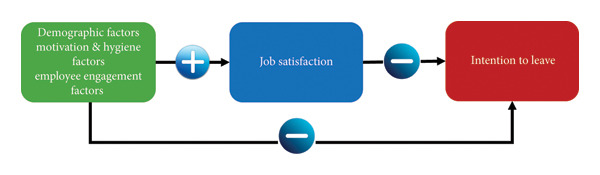
Conceptual framework for nursing turnover intention.

Despite growing global attention to turnover in healthcare, few studies have examined how demographic characteristics, job satisfaction and work environment influence staff intention to leave the nursing home settings in Singapore. Most existing literature has focused on acute care or hospital‐based nurses, with limited attention given to unlicensed assistive personnel in long‐term care. As Alenazy et al. [9] highlighted, understanding these influencing factors is essential for designing targeted, organisation‐specific retention strategies. Hence, this study sought to address this gap by exploring the predictors of intention to leave among nursing home care staff, guided by a conceptual framework (Figure 1) that integrates Herzberg’s Two‐Factor Theory [12] and the model by Smokrović et al. [13]. The proposed conceptual framework distinguished between individual attributes (e.g., demographics and job roles), intrinsic motivators (e.g., recognition, career advancement and meaningful work) and extrinsic hygiene factors (e.g., supervision, staffing adequacy, organisational policies and salary). It suggested that imbalances among these domains can lead to negative staff outcomes, including dissatisfaction and increased turnover intention.

## 2. Research Methodology

### 2.1. Research Questions

1. What is the overall level of intention to leave among nursing home care staff?
2. Do demographic characteristics (e.g., age, gender, years of experience and education) significantly influence the intention to leave among nursing home care staff?
3. What are the predicting factors that significantly influence the intention to leave among nursing home care staff?

### 2.2. Design

The study employed a cross‐sectional quantitative design using survey methodology to gather data on participants’ perceptions, which was useful for the researchers to gather a broad range of opinions to understand the phenomenon [14]. The study protocol was approved by the Charisma University Ethics Committee (CU20243/100423).

### 2.3. Study Sample and Settings

The study was conducted at two nursing homes in Singapore, which serve an ageing population with concomitant chronic diseases. There was a total of 273 care staff (comprising 56 registered nurses, 15 enrolled nurses, 85 nursing aides and 117 healthcare assistants) in the two facilities, including full‐time and part‐time staff. The study was conducted over a 6‐month period between January and June 2024. The population studied in the research study included all care staff working in the two nursing homes. Using the Raosoft calculator, a minimum sample size of 160 participants was targeted for the quantitative aspect of the study using a survey based on a 5% margin of error and 95% confidence level to achieve internal validity [14]. A total of 167 valid responses were collected using convenience sampling technique, yielding a 61.2% response rate. The final sample was deemed representative, as the proportions of staff roles among respondents approximated those in the overall staff population at the two nursing homes.

### 2.4. Instruments

Quantitative data were collected using an adapted structured questionnaire, which combined three established instruments: the Practice Environment Scale of the Nursing Work Index (PES‐NWI) [15], the Intention to Leave Scale (ILS) [16] and the Job Satisfaction Survey (JSS) [17]. This comprehensive instrument also included items to collect demographic information specific to nursing home staff.

### 2.5. Data Collection

The data collection process was conducted between January and February 2024. Eligible participants were invited via email or during handover sessions to complete an online questionnaire, which took an average of 20–30 min to complete. Participants were given information about the study, and written informed consent was taken before the data collection. Participation was entirely voluntary. The data collected would immediately be entered into the organisation’s electronic database.

Spector’s [18] ILS was used to measure staff intention to leave the organisation because of its established reliability, validity and widespread use in organisational psychology and management research. It consists of three items. Participants responded to each item using a six‐point Likert scale, ranging from 1 (*strongly disagree*) to 6 (*strongly agree*). The total score is calculated by summing the responses to all three items, resulting in a possible score range from 3 to 18. Higher scores indicate a greater intention to leave the current job [18]. In terms of reliability and validity, the ILS has demonstrated high internal consistency reliability, with Cronbach’s alpha values reported between 0.84 and 0.91 across various studies [19]. Its validity has been confirmed through correlations with actual employee turnover in longitudinal studies. The three‐item scale is preferred over a single‐item measure due to its greater reliability and predictive power. The scale explains approximately 70% of the variance in turnover intentions, making it a robust predictor of potential employee turnover [20].

The JSS developed by Spector [17] was chosen as a measure of job satisfaction due to its established reliability and validity. The instrument’s widespread use in diverse organisational settings allows for comparison of similar studies across different healthcare setting. The JSS is a 36‐item scale that measures nine facets of job satisfaction: (i) pay, (ii) opportunities for promotion, (iii) quality of supervision, (iv) fringe benefits, (v) performance‐based rewards, (vi) work environment, (vii) relationships with coworkers, (viii) the nature of the work and (ix) communication within the organisation. Each facet is assessed using four items, and participants respond using a six‐point Likert scale ranging from “*strongly disagree*” to “*strongly agree*”. Possible scores range from 36 to 216, with higher scores indicating greater job satisfaction. The JSS has demonstrated strong psychometric properties across various studies. Spector [17] reported internal consistency reliabilities (coefficient alpha) ranging from 0.60 for the coworker subscale to 0.91 for the total scale. Subsequent studies have consistently found similar reliability coefficients, supporting the instrument’s reliability [21].

The PES‐NWI was used to measure the nursing practice environment in this study. Developed by Lake [15], the PES‐NWI is the most widely reported and endorsed measure for assessing the state of the work environment for nurses and care staff globally [22]. The PES‐NWI consists of 31 items divided into five subscales: (i) nurse participation in hospital affairs; (ii) nursing foundations for quality of care; (iii) nurse manager ability, leadership and support of nurses; (iv) staffing and resource adequacy and (v) collegial nurse–physician relations [15]. Each item is rated on a four‐point Likert‐type scale, ranging from 1 (*strongly disagree*) to 4 (*strongly agree*), with higher scores indicating a more favourable practice environment [23]. The PES‐NWI has demonstrated strong psychometric properties across various healthcare settings and countries [24]. Its validity has been established through extensive use in research and endorsement by quality‐promoting organisations such as the National Quality Forum and the Joint Commission [22]. The instrument has shown high reliability, with Cronbach’s alpha coefficients typically ranging from 0.71 to 0.84 for the five subscales [15, 23]. The PES‐NWI has been applied in numerous clinical practice settings, including intensive care units, medical‐surgical units, outpatient clinics and speciality areas such as oncology and psychiatry [24]. It has been used in both acute and chronic care environments, as well as in various countries, demonstrating its versatility and cross‐cultural applicability [22]. The instrument’s widespread use allows for benchmarking and comparison across different healthcare organisations and countries [24]. It has been employed in studies examining the relationship between practice environment and various outcomes, including nurse job satisfaction, burnout, patient safety and quality of care [23]. For this study, we used the original 31‐item version of the PES‐NWI to ensure comparability with the existing literature and to maintain the instrument’s established validity and reliability. The scale was administered to nursing staff as part of a larger survey assessing various aspects of the work environment and job satisfaction.

### 2.6. Data Analysis

The data analysis was conducted using IBM Statistical Package for the Social Sciences (SPSS) 29.0. Descriptive statistics were used to summarise the demographic characteristics and key variables, while inferential statistics were used to examine the relationships between demographic characteristics, work environment, job satisfaction and intention to leave.

## 3. Results

### 3.1. Demographic Characteristics

The sample consisted of 167 care staff working in two nursing homes in Singapore. The demographic characteristics of the participants are summarised in Table 1. The demographic characteristics of the study participants (*n* = 167) reveal a diverse workforce in terms of age, gender, years of experience, job role and education level. The participants were predominantly in their 30s, representing 33% of the sample, followed by those in their 40s at 30%. Most participants were female, comprising 70% of the sample. Most participants had 1–5 years of experience (54%), with fewer individuals having over 10 years of experience. In terms of job roles, healthcare assistants constituted the largest group at 47%, followed by nursing aides (28%) and registered nurses (18%). Educational qualifications varied, with 36% holding a diploma, while a smaller proportion possessed Bachelor’s or Master’s degrees.

**Table 1:** Demographic characteristics (*n* = 167).

Variable	Frequency	Percentage
Age		
20–29	40	24
30–39	55	33
40–49	50	30
50 and above	22	13
Gender		
Male	50	30
Female	117	70
Years of experience		
1–5 years	90	54
6–10 years	65	39
11–15 years	10	6
16 years and above	2	1
Job role		
Registered nurse	30	18
Enrolled nurse	12	7
Nursing aide	47	28
Healthcare assistant	78	47
Education level		
Certificate and below	40	24
Diploma	60	36
Bachelor’s degree	50	30
Master’s degree and above	10	6
Others	7	4

### 3.2. Research Question 1—Overall Intention to Leave Among Nursing Home Care Staff

Table 2 presents the descriptive statistics for the main variables of interest: JSS scores, ILS and work environment score (PES‐NWI). The work environment score (mean = 79.51, SD = 8.487) reveals a wide range of perceptions, with some participants viewing their environment as supportive, while others have less favourable views, highlighting diverse experiences among the nursing staff. The job satisfaction score (mean = 101.87, SD = 10.138) suggests moderate to high satisfaction levels among participants, though the standard deviation suggests a significant variability in satisfaction among the participants. The intention to leave score (mean = 9.18, SD = 2.494) reflects a moderate overall intention to leave, with some participants showing a higher propensity for turnover intention.

**Table 2:** Descriptive statistics for variables.

Variable	*N*	Mean	Std. deviation	Minimum	Maximum
PES‐NWI	167	79.51	8.487	36	90
JSS	167	101.87	10.138	76	119
ILS	167	9.18	2.494	3	15

### 3.3. Research Question 2—Impact of Demographic Characteristics on the Intention to Leave

Table 3 reveals significant demographic differences in intention to leave among care staff. Female employees had higher scores (*M* = 9.69, SD = 2.37) than males (*M* = 7.98, SD = 2.40), indicating possible gender‐specific challenges. Age differences showed the 30–39 group with the highest scores (*M* = 10.20, SD = 2.28), while the 20–29 group had the lowest (*M* = 7.43, SD = 2.04), suggesting that mid‐career staff may experience more turnover pressure or extrinsic factors encouraging their intent to leave. Job role differences were notable, with nursing aides reporting the highest intention to leave (*M* = 10.15, SD = 2.22), while registered nurses had the lowest (*M* = 7.57, SD = 2.10). Education level also influenced intention to leave, with diploma holders showing the highest scores (*M* = 10.30, SD = 2.30). This suggests that this group may feel a stronger need for career progression and perceive limited opportunities in their current roles.

**Table 3:** Demographic differences in intention to leave (ILS).

Demographic variable	N	Mean	SD	F‐statistic/*t*‐test	*p* value
**Age**				13.871	< 0.001^∗∗^
20–29	40	7.43	2.037		
30–39	55	10.20	2.280		
40–49	50	9.78	2.410		
50 and above	22	8.45	2.132		
**Gender**				*t* = −4.269	< 0.001^∗∗^
Male	50	8.0	2.4		
Female	117	9.7	2.4		
**Years of experience**				1.583	NS
1–5 years	90	9.0	2.6		
6–10 years	65	9.6	2.4		
11–15 years	10	8.0	2.3		
16–20 years	2	9.0			
> 20 years	0	0	0		
**Job role**				9.132	< 0.001^∗∗^
Registered nurse	30	7.6	2.1		
Enrolled nurse	12	7.8	2.9		
Nursing aide	47	10.2	2.2		
Healthcare assistant	78	9.4	2.4		
**Education level**				11.378	< 0.001^∗∗^
Certificate and below	40	7.4	2.0		
Diploma	60	10.3	2.3		
Bachelor’s degree	50	9.6	2.3		
Master’s degree and above	10	8.1	2.1		
Others	7	8.1	1.9		

*Note:* Values are presented as mean (standard deviation). NS = not significant (*p*  >  0.05, ^∗^ *p*  <  0.05 and ^∗∗^ *p*  <  0.01).

### 3.4. Research Question 3—Predictors of the Intention to Leave Among Nursing Home Care Staff

A hierarchical multiple regression was used to determine the impact of demographic variables, nursing work environment (PES‐NWI) and job satisfaction (JSS) on intention to leave scores (ILS). The demographic variables entered in Model 1 included age, gender, years of experience, job role, and education. In Model 2, PES‐NWI was added, and in Model 3, JSS was included as a mediator (Table 4).

**Table 4:** Multiple linear regression for predictors of intention to leave.

Model	Predictor	*R*	*R* ^2^	Std. Error	F	*p*
1	Demographics (age, gender, years of experience, job role and education)	0.403	0.162	2.318	6.229	< 0.001
2	Demographics + PES‐NWI	0.436	0.190	2.316	3.655	< 0.001
3	Demographics + PES‐NWI + JSS	0.488	0.238	2.313	2.419	0.002

In Model 1 (demographics only), the model explained 16.2% of the variance in ILS (*R* ^2^ = 0.162), which was significant (*F* [5,161] = 6.229, *p* < 0.001). On subanalysis for Model 1, none of the demographic variables were significant predictors of intention to leave. In Model 2, which added work environment factors (e.g., nurse participation, nursing foundations, nurse manager ability, staffing and resource adequacy and collegial relations), the model fit significantly improved (*F* [10, 156] = 3.655, *p*  <  0.001) and was able to explain 19.0% of the variance in intention to leave. Among the demographic variables, only the “job role” emerged as a significant predictor of intention to leave. The results indicated that the job role had a significant negative relationship with ILS (*β* = −0.446, *p* = 0.033), supporting the findings in Research Question 2, whereby certain job roles, specifically the nursing aides, were more likely to express an intention to leave than others. Among the work environment factors, only the “Staffing and Resource Adequacy” subcomponent of PES‐NWI was statistically significant (*β* = −0.153, *p* = 0.042), suggesting that better staffing and resource adequacy might be associated with lower intention to leave. None of the other PES‐NWI subcomponents were significant predictors of ILS.

Finally, in Model 3, job satisfaction factors (e.g., pay, promotion, supervision, performance‐based rewards, coworker relations, nature of work and communication) were added, further enhancing the model’s fit (*F* [19, 147] = 2.419, *p* = 0.002), explaining 23.8% of the variance in intention to leave. The subanalysis of the final model shows that only two job satisfaction factors, supervision and staffing/resource adequacy, were significant predictors of intention to leave. Both factors highlight the importance of supportive management and adequate resources in reducing turnover intentions within nursing environments. In contrast, demographic factors and other job satisfaction subcomponents had a minimal effect on predicting ILS.

## 4. Discussion

The study revealed that nursing home care staff in Singapore reported a moderate overall intention to leave, which suggested some level of dissatisfaction with their current employment. This aligns with previous studies highlighting staff turnover as a persistent challenge in nursing homes globally, particularly in environments where job demands are high and resources are often insufficient [5, 7, 11]. Multivariable models further identified inadequate staffing, resources and low supervisory support as the strongest predictors of turnover intention.

### 4.1. Impact of Demographic Characteristics on Intention to Leave

Our findings concur with Alenazy et al. [9] who found that demographic factors does influence turnover intentions in long‐term care, specifically for females, mid‐career staff and nursing aides. For example, female care staff reported higher intention to leave than males, suggesting that caregiving responsibilities and work‐life conflicts disproportionately affect women [7, 11, 25, 26]. Mid‐career staff (those aged 30–39 years) expressed the greatest turnover intention, which can be attributed to career stagnation, burnout or dissatisfaction from a lack of career advancement [5, 27]. Among the job grades, nursing aides showed the highest turnover intentions, mirroring several studies which found that this frontline group faced the bulk of physical and emotional labour [7, 28, 29]. Other contributing factors could include the underutilisation of their qualifications or limited career and educational advancement pathways in the local healthcare scene [27, 28, 30, 31].

### 4.2. Predictors of Intention to Leave

The regression analysis provided further insights into the factors predicting turnover intentions. In Model 2, work environment factors were added, and “Staffing and Resource Adequacy” emerged as a significant predictor of intention to leave. This finding underscores the importance of adequate staffing levels and resources in reducing turnover intentions. When staff feel that resources are inadequate or that staffing is insufficient to meet the job demands, they are more likely to experience burnout, dissatisfaction and, eventually, a desire to leave [27, 32].

In the final model, job satisfaction factors (supervisory support and staffing/resource adequacy) emerged as significant predictors of turnover intention. However, the modest *R* ^2^ value of 0.238 suggested many unexplained variables that could have also influenced turnover intentions. This result echoes those found in other studies [31, 33] which underscore the notion that staff turnover intention might be influenced by interplay of multifaceted variables, including external labour market conditions and societal views towards work commitment.

Despite the unexplained variance, our study highlighted the crucial role that supportive management plays in influencing employee retention in Singapore’s long‐term care setting. Staff who perceive strong managerial support experience lower burnout and higher job satisfaction. These findings concur with other studies which suggest that supervisory support can buffer the negative effects of work stress and improve job satisfaction [13, 30]. Moreover, demographic factors showed minimal impact in the final model, suggesting strategies to improve job satisfaction using targeted interventions, such as supervisor training and conducting staff feedback sessions, can significantly reduce turnover intention [34, 35].

### 4.3. Implications for Practice

This study has important implications for nursing home management in Singapore. Firstly, targeted interventions to improve staffing levels and resource availability could significantly reduce turnover intentions, particularly among nursing aides and mid‐career staff. Additionally, offering career progression opportunities, especially for diploma holders and nursing aides, may mitigate turnover by providing staff with a clear career and educational advancement pathway [29]. Secondly, improving managerial support through enhanced supervisor training could help create a more supportive work environment, which is crucial for reducing staff turnover. Organisations may benefit from implementing mentorship programs, leadership training and initiatives to improve communication and support between managers and staff [34, 35]. Lastly, addressing gender‐specific challenges, particularly for female staff who reported higher turnover intentions, could involve creating flexible work schedules or providing more support for work‐life balance. Gender‐responsive policies that acknowledge and address these unique challenges may help reduce the higher turnover intention observed among female employees [25].

### 4.4. Limitations and Future Research

There are some limitations to this study. First, the cross‐sectional design limits the ability to draw causal conclusions about the relationships between the variables studied. Longitudinal research could provide more insight into how these factors influence turnover over time. Second, while the study was conducted in two nursing homes in Singapore, the findings may need to be generalisable to other nursing homes or healthcare settings, particularly those outside Singapore. Third, the sample representativeness across job categories and work shifts was not determined, and we did not employ stratified sampling method. This design limitation might introduce study bias if certain roles or shifts are underrepresented. Fourth, the use of self‐administered questionnaires may have introduced self‐report bias due to the social desirability of some respondents to under‐report, as turnover intention can be considered a socially sensitive topic. Future research could expand the scope to include a broader range of nursing homes and other long‐term care settings to provide a more comprehensive understanding of turnover intentions among care staff.

Future research could also explore qualitative methods to complement the quantitative findings from this study. Conducting interviews or focus groups with care staff may provide more in‐depth insights into the specific factors contributing to their intention to leave and could help identify additional factors that were not captured in the quantitative survey. Additionally, future studies could examine the effectiveness of interventions to reduce turnover, such as improving staffing levels, enhancing managerial support or providing career development opportunities.

## 5. Conclusion

This study provides valuable insights into the overall intention to leave among nursing home care staff in Singapore and highlights the significant role of job satisfaction and work environment factors in influencing turnover intentions. By addressing staffing adequacy, managerial support and career development issues, nursing homes can create a more supportive and satisfying work environment, ultimately reducing turnover and improving the quality of care provided to residents.

## Disclosure

The final manuscript reflects the authors’ own intellectual work and accountability.

## Conflicts of Interest

The authors declare no conflicts of interest.

## Author Contributions

All the authors performed the role of conceptualisation, data curation, formal analysis, methodology, validation and review. The revised manuscript was critically reviewed and fact‐checked by Hongli Sam Goh and Mun Leong Tang to ensure its accuracy, originality and compliance with ethical and academic standards.

## Funding

This research did not receive any grant from any funding agency in the public, commercial or not‐for‐profit sectors.

## Data Availability

The data that support the findings of this study are available on request from the corresponding author. The data are not publicly available due to privacy or ethical restrictions.
